# Feeding and resting behaviour of malaria vector, *Anopheles arabiensis *with reference to zooprophylaxis

**DOI:** 10.1186/1475-2875-6-100

**Published:** 2007-07-30

**Authors:** Aneth Mahande, Franklin Mosha, Johnson Mahande, Eliningaya Kweka

**Affiliations:** 1 KCM College of Tumaini University, PO BOX 2240, Moshi, Tanzania; 2 Ifakara Health Research and Development Centre, Department of Public Health Entomology, PO BOX 53, Ifakara-Morogoro, Tanzania; 3 Kilimanjaro Christian Medical Center Ophthalmology Department, PO BOX 2240, Moshi Tanzania

## Abstract

**Background:**

The most important factor for effective zooprophylaxis in reducing malaria transmission is a predominant population of a strongly zoophilic mosquito, *Anopheles arabiensis*. The feeding preference behaviour of Anopheline mosquitoes was evaluated in odour-baited entry trap (OBET).

**Methods:**

Mosquitoes were captured daily using odour-baited entry traps, light traps and hand catch both indoor and in pit traps. Experimental huts were used for release and recapture experiment. The mosquitoes collected were compared in species abundances.

**Results:**

*Anopheles arabiensis *was found to account for over 99% of *Anopheles *species collected in the study area in Lower Moshi, Northern Tanzania. In experimental release/capture trials conducted at the Mabogini verandah huts, *An. arabiensis *was found to have higher exophilic tendency (80.7%) compared to *Anopheles gambiae *(59.7%) and *Culex spp*. (60.8%). OBET experiments conducted at Mabogini collected a total of 506 *An. arabiensis *in four different trials involving human, cattle, sheep, goat and pig. Odours from the cattle attracted 90.3% (243) compared to odours from human, which attracted 9.7% (26) with a significant difference at P = 0.005. Odours from sheep, goat and pig attracted 9.7%, 7.2% and 7.3%, respectively. Estimation of HBI in *An. arabiensis *collected from houses in three lower Moshi villages indicated lower ratios for mosquitoes collected from houses with cattle compared to those without cattles. HBI was also lower in mosquitoes collected outdoors (0.1–0.3) compared to indoor (0.4–0.9).

**Conclusion:**

In discussing the results, reference has been made to observation of exophilic, zoophilic and feeding tendencies of *An. arabiensis*, which are conducive for zooprophylaxis. It is recommended that in areas with a predominant *An. arabiensis *population, cattle should be placed close to dwelling houses in order to maximize the effects of zooprophylaxis. Protective effects of human from malaria can further be enhanced by keeping cattle in surroundings of residences.

## Background

Host-odours play a major role in the orientation of nocturnal mosquitoes towards their hosts [1]. Differences in host-preference between mosquito species are, therefore, likely to be reflected in their response to different host odours offered [2]. Carbon dioxide is a major component of the breath of all warm-blooded vertebrates and has been studied intensively for its attractiveness to mosquitoes [3,4]. A number of studies have confirmed the role of CO_2 _in the host-seeking behaviour of the highly anthropophilic *Anopheles gambiae *s.s [4].

Members of the *An. gambiae *complex are important malaria vectors in sub-Saharan Africa, but these species differ strongly in host-preference [5] which is assumed to be stimulated by the odour produced by the host [6]. *Anopheles arabiensis *occupies over 70% of sub-Saharan Africa; the species dominates in arid zones and some of highland areas [7,8] and adapts to endophagic and endophilic patterns, where hosts are domestic and indoor, but adopts exophagic patterns where hosts are mainly outdoors. In response to indoor spraying, they become completely exophilic [9-11].

There have been reports of instances where the introduction of livestock has apparently reduced prevalence of the disease, the reduction in malaria that occurred in Europe and in United States earlier last century has been attributed partly to the increase in livestock numbers [12].

*Anopheles arabiensis *has a low Human Blood Index (HBI) and shows a marked preference for cattle and other warm-blooded animals [13]. It has a high degree of zoophily in Madagascar as demonstrated by HBI reported from various environmental settings [8,14,15]. Lower proportion of human blood meals (26%) were recorded in *An. arabiensis *collected from sites where cattle were kept closer to human housing than in those collected from sites where cattle were kept some distance from humans (57%)[16,17]

**Figure 1:**
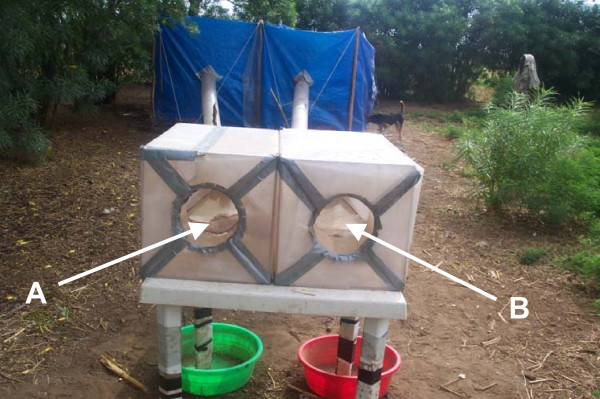
Odour Baited Entry Traps (OBETs) from the tents with different baits. Trap A with a cattle and B with human, both of the same weight.

The behaviour of *An. arabiensis *was assessed in this paper by using three different ways. First by using experimental huts, where the resting behaviour of *An. arabiensis *was assessed and compared with other species common in the community (*An. gambiae *and *Culex quinquefasciatus*). Second by using odour-baited entry traps: OBETs (see figure 1) involving humans and four different animals usually kept in the community (cattle, goats, sheeps and pigs). Mosquito behaviour was also assessed by using HBI comparing the feeding behaviour of mosquitoes in three different communities.

This paper describes the results of a study of the response of *An. arabiensis *to humans, cattle, goat, sheep and pig in lower Moshi, Northern Tanzania.

## Methods

### Study Area

Mabogini, Rau and Mtakuja villages were selected for this study. These villages are in Lower Moshi, Northern Tanzania, at an altitude of about 800 m above sea level within Maasai savannah at the foothills of Mount Kilimanjaro. Four houses in each selected village were randomly selected for Light Trap Catches (LTC) and Pyrethrum Spray Catches (PSC). OBET experiments were also conducted close to a dwelling house at Mabogini, Lower Moshi area.

### Animals for experiments

Animals were taken from the villagers who volunteered during the experiments after the consent procedures.

### Assessment of *An. arabiensis *resting habit

This was done by assessing exophilic tendencies of *An. arabiensis *in comparison with *An. gambiae *s.s and *Culex quinquefasciatus*. The huts were a slight modified from those of Verandah Trap Huts (VTH) described by Smith[18].

### Assessment of *An. arabiensis *host preference

This was done by estimation of relative attractiveness of man and livestock to *An. arabiensis*. The technique was designed to simulate natural condition as far as possible. This experiment was done in two phases as follows:-

### a) Odour Baited Entry Traps (OBETs)

The experimental arrangement was similar to that of Costantini *et al *[19] and Duchemin *et al *[20]. Two OBETs, designed to catch host-seeking mosquitoes responding mainly to odour cues, were placed next to one another near to a residential compound at Mabogini village. The OBETs were similar to lobster-pot entry traps and baited with test host. Odours were drawn from reservoir bait in a tent to the trap by a fan. They were set approximately 1.5 m high on wooden tables. Air coming from two tents standing approximately 7 m upwind of the traps was drawn into the OBETs by fans via plastic air ducts. Mosquitoes had a choice of odours from two alternative hosts presented to the approaching mosquitoes.

One adult man and a calf of similar mass were concealed in two separate tents and their odour drawn by fans to the OBETs via inflatable 'lay-flat' polythene tubing. The calf, a zebu breed, was tethered inside a small fence and covered with the polyethylene tent. On any trapping night, the OBETs were operated from 19.00 to 05.00 a.m. After every two days the traps were exchanged from side to side in order to compensate for any positional effects. Subsequently, other domestic animals were also placed in the two different tents where mosquitoes preferences were assessed between a calf (50 kg) and three goats (15 kg each), three sheeps (15 kg. each) and lastly three pigs (15 kg each).

### b) Estimation of Human Blood Indices (HBI) of *An. arabiensis *collected from three different villages

The pyrethrum spray-catch method is fully described in the WHO entomology manual [21]. In each house, a bedroom that was occupied by one sleeper was selected for the mosquitoes collection. Out of the selected four houses in each village, two had livestock and other two had no livestock.

Outdoor resting mosquitoes were collected by standard methods involving pit traps and empty drums [22]. Four pit traps were constructed in each village and four tanks drums placed in each village. The pyrethrum spray catch was done by covering the floor and furniture in bedrooms with white sheets. The room was then sprayed with pyrethrum (0.4% volume diluted in kerosene). After 10 minutes, the knocked-down mosquitoes were collected from the white sheets as described in WHO entomology manual [21] and Premasiri *et al *[23].

Light trap collection was done by suspending a light trap with its base at 45 cm above the head of a person sleeping under intact bednet. Light trap was operated from 18:30 to 06:30. Collection of mosquitoes from the trap was performed as described [21]. Identification of blood meal source was carried out according to methods described by Bray *et al *[24].

### Data analysis

The data entry was done in Microsoft Excel 2000 and analysis was carried out using statistical package for social science (SPSS) version 10 programme. The significance test was estimated assuming an α (two sided) = 0.05). Other data were analysed by using EpiInfo™ Version 3.2.2 programme where χ^2 ^and P value were calculated.

### Ethical considerations

Before conducting this study, ethical clearance was sought from Kilimanjaro Christian Medical College Research Ethics Committee. Permission from the district and respective village authorities was obtained. Both verbal and written informed consent was obtained from the head of the households selected for the study.

## Results

During the study in experimental huts the exophily pattern and feeding behaviour of *An. arabiensis *was assessed.

### *An. arabiensis *resting habits

A total of 930 mosquitoes were released into the experimental hut. Out of these, 81 were lost (could not be recovered) during the experiment and were removed from the analysis. Therefore, 849 mosquitoes were used in the data analysis. Of these 31.8% (270) were *An. arabiensis*, 35.1 % (298) were *An. gambiae s.s *and 33.1 % (281) were *Culex spp*.

**Figure 2:**
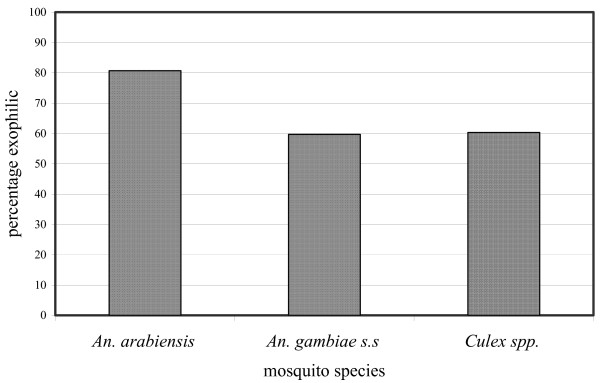
The exophilic behaviour of three mosquito species in experimental huts.

These results have shown that, *An. arabiensis *had higher exophilic tendency (80.7%) compared to *An. gambiae *s.s (59.7%) and *Culex spp *(60.8%) as in Figure 2. The difference was statistically significant (χ^2 ^= 23, P = 0.001).

### OBETs experiments

During the period of this study, *An. arabiensis *was the predominant species (79.5%) followed by *Culex quinquefasciatus *as shown in Table 1. *Anopheles funestus *accounted for 0.55% of the outdoor and indoor collected mosquitoes.

**Table 1:** Mosquitoes collected from indoor (hand catch) and outdoors (pit trap) methods during the OBETs experiment in the study area.

Specie type	Method of collection	Number	percentage
	Indoor	184	25.7
An. arabiensis	Outdoor	533	74.3
	Total	717	
	Indoor	5	100
An. funestus	Outdoor	0	0
	Total	5	
Culex spp.	Indoor	41	22.8
	Outdoor	139	77.2
	Total	180	
Total		902	

**Figure 3:**
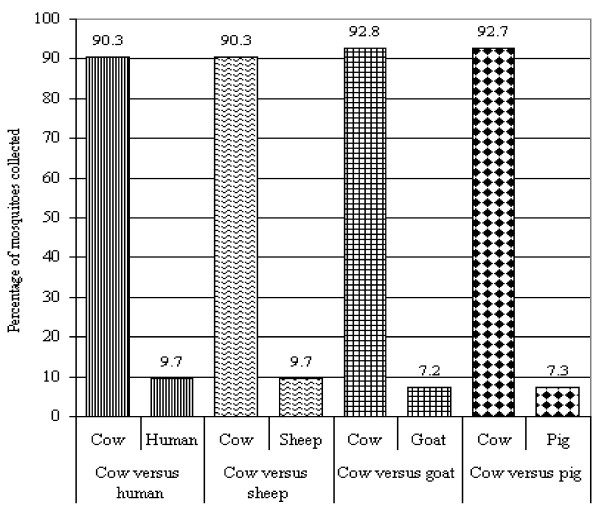
*Anopheles arabiensis *collected from OBETs with human and different animals.

The OBETs collected a total of 506 female *An. arabiensis *in four different experiments (where different baits in separate tent were compared to cattle). The greatest numbers of mosquitoes were collected from cattle traps compared to human, goat, pig and sheep traps (Figure 3).

#### Experiment I: Cattle versus human

Two hundred and sixty nine female *An. arabiensis *mosquitoes were collected from the two traps. Of these 90.3 % (243) were collected from the cattle odour trap and 9.7 % (26) from human odour trap.

#### Experiment II: Cattle versus sheep

Thirty-one *An. arabiensis *mosquitoes were collected. Only 9.7 % (3) were collected in the sheeps odour trap and the rest 90.3 % (28) were collected in the cattle odour trap.

#### Experiment III: Cattle versus goat

A total of 83 *An. arabiensis *mosquitoes were collected. Higher proportion (92.8%) was collected from cattle trap compared to that collected from goat trap (7.2%).

#### Experiment IV: Cattle versus Pig

One hundred and twenty three *An. arabiensis *were collected in cattle and pigs traps. Of these 92.7 % (114) and 7.3 % (9) were collected from cattle and pig trap respectively.

### Results from experimental hut showing feeding preference

Zoophilic tendency of *An. arabiensis *mosquito was assessed during the host rotation in the experimental hut where treated cattle, untreated cattle and human were rotated in the huts. More blood-fed mosquitoes were collected from hut with untreated cattle (Mean = 22.5) than from hut with human sleepers (mean = 9.0).

#### Human blood indices

A total of 3,902 mosquitoes were collected from indoors using pyrethrum spray-catch, outdoors using pit trap and empty drums in study villages, namely Mabogini, Rau and Mtakuja. Among these 1,792 were *An. arabiensis*, 2,093 were *Culex spp*. and 17 were *An. funestus*. Of the collected *An. arabiensis *mosquitoes only 417 were blood-fed and were tested for Human Blood Index (HBI).

The results indicated that in all of the three villages, lower HBI were observed in mosquitoes collected indoors (0.4–0.7) and outdoor (0–0.1) from households with cattle compared to those households with no cattle.

## Discussion

The experimental hut studies confirm that *An. arabiensis *has a tendency to escape from houses after feeding, a behavioural pattern normally referred to as exophily. This behaviour was also shown by Smith[25] in the Umbugwe area (now called Magugu) of Northern Tanzania. The exophily rate for *Cx. quinquefasciatus *(60.8%) and *An. arabiensis *(80.7%) observed in this study is similar to observations made by Kulkarni *et al *[26]. Percentage of mosquitoes which escaped through the eaves and windows of the experimental huts on the following morning after entry into the control huts were 70.9% (*n *= 3,664) *An. arabiensis *compared to 66.0% (*n *= 2,075) *Cx. quinquefasciatus*. Elsewhere in East Africa, Highton *et al *[27] reported that *An. arabiensis *in the Kisumu area, Kenya, showed a tendency to occur outdoors 2.2 times more frequently than indoors, while Joshi *et al *[28] reported 2.8 times. White *et al *[5] observed that in Segera, Tanzania, *An. arabiensis *displayed a 2.3 times greater tendency to occur outdoors compared to *An. gambiae s.s*.

The exophilic behaviour demonstrated by *An. arabiensis *should be taken into consideration when planning control strategies. Residual house spraying will, therefore, have little impact in areas with a predominant *An. arabiensis *population since the targeted vector will not spend enough time on sprayed walls to pick up the lethal insecticide dose.

*Anopheles arabiensis *was found to be the predominant mosquito species at lower Moshi, accounting for 79.5% of the total mosquito population and 99.3% of Anopheline species. The study villages are in semi-arid belt and similar observations have been reported previously from the same area [29] and elsewhere in Africa [5,30,31]. The OBETs experiments showed a very strong attraction of *An. arabiensis *to cattle odour. When compared to human and other livestock such as sheep, pig and goat, the cattle attracted over 90% of the collected *An. arabiensis*. Similar findings with the OBETs have also been reported by Duchemin *et al *[20] in Madagascar and by Diatta *et al *[32] in Senegal. The unattractive natures of odour from goat and pig have not been reported before.

Strong zoophilic tendencies of *An. arabiensis *have also been observed elsewhere including Mwea irrigation area in Kenya [8]. The present studies have demonstrated the protective effect of cattle against mosquito bites. A lower proportion of *An. arabiensis *collected from houses with cattle were found to have fed on humans as indicated by low HBI compared to houses without cattle. Similar findings have been reported elsewhere [8,14,33,34]. These results contradict reported observations that proximity of cattle to humans increases mosquito bites on humans [13,35,36]. This could have been due to differences in species and environmental conditions in those areas.

In this study area, the OBETs and experimental hut studies as well as HBI community observations provide strong evidence that cattle kept around dwelling houses are effective at offering protection against *An. arabiensis *bites and consequently reduces malaria incidences. This is regardless of other factors such as cattle-human ratio and proximity of animals to mosquito breeding sites as postulated by Saul [37].

## Competing interests

The author(s) declare that they have no competing interests.

## Authors' contributions

AMM and FWM Co-designed the study, participated in analysis and interpretation of data and contributed to the drafting of the manuscript. JMM carried out the analysis of mosquitoes assisted with data analysis and interpretation and was involved in the drafting of the manuscript. EJK coordinated the study, participated in the analysis and interpretation of results, and designed the first draft and final version of the manuscript and critically evaluation thereof. All authors read and approved the final manuscript.
